# Mesoporous MgTa_2_O_6_ thin films with enhanced photocatalytic activity: On the interplay between crystallinity and mesostructure

**DOI:** 10.3762/bjnano.3.13

**Published:** 2012-02-13

**Authors:** Jin-Ming Wu, Igor Djerdj, Till von Graberg, Bernd M Smarsly

**Affiliations:** 1 Department of Materials Science and Engineering, Zhejiang University, Hangzhou 310027, China; 2 Ruđer Bošković Institute, Bijenička 54, 10000 Zagreb, Croatia; 3 University of Giessen, Institute of Physical Chemistry, Heinrich-Buff-Ring 58, D-35392 Giessen, Germany

**Keywords:** magnesium tantalate, mesoporous materials, photocatalytic activity, self-assembly, thin films

## Abstract

Ordered mesoporous, crystalline MgTa_2_O_6_ thin films with a mesoscopic nanoarchitecture were synthesized by evaporation-induced self-assembly (EISA) in combination with a sol–gel procedure. Utilization of novel templates, namely the block copolymers KLE (poly(ethylene-*co*-butylene)-*b*-poly(ethylene oxide)) and PIB6000 (CH_3_C(CH_3_)_2_(CH_2_C(CH_3_)_2_)_107_CH_2_C(CH_3_)_2_C_6_H_4_O(CH_2_CH_2_O)_100_H), was the key to achieving a stable ordered mesoporous structure even upon crystallization of MgTa_2_O_6_ within the mesopore walls. The effect of the calcination temperature on the ability of the mesoporous films to assist the photodegradation of rhodamine B in water was studied. As a result, two maxima in the photocatalytic activity were identified in the calcination temperature range of 550–850 °C, peaking at 700 °C and 790 °C, and the origin of this was investigated by using temperature-dependent X-ray scattering. Optimal activity was obtained when the mesoporous film was heated to 790 °C; at this temperature, crystallinity was significantly high, with MgTa_2_O_6_ nanocrystals of 1.6 nm in size (averaged over all reflections), and an ordered mesoporous structure was maintained. When considering the turnover frequency of such photocatalysts, the optimized activity of the present nanoarchitectured MgTa_2_O_6_ thin film was ca. four times that of analogous anatase TiO_2_ films with ordered mesopores. Our study demonstrated that high crystallinity and well-developed mesoporosity have to be achieved in order to optimize the physicochemical performance of mesoporous metal-oxide films.

## Introduction

Because of its excellent microwave dielectric properties, MgTa_2_O_6_ is one of the ternary oxides that have been well studied for application as dielectric resonators operating at microwave frequencies [1–9]. In the form of thin films, it is also considered to be one of the most promising candidates for use in polarizers in optical communications and other optical devices [5]. The traditional ceramic method of synthesizing MgTa_2_O_6_, i.e., solid-state reaction, requires high temperatures of 1200–1400 °C, which produces coarse grains and an inhomogeneous composition [6–8]. Crystalline MgTa_2_O_6_ powders were fabricated at 850 °C by a molten-salt method and at 550 °C by a wet-chemical approach [9]. Thin films containing mainly MgTa_2_O_6_ and Mg_4_Ta_2_O_9_ phases can be formed by reactions between MgO(001) substrates and Ta–O vapors, which are produced by electron-beam evaporation of a Ta_2_O_5_ powder target in a high-vacuum system, at temperatures of 700–1000 °C [5].

In 1998, Kato and Kudo reported that MgTa_2_O_6_ that had been synthesized by calcination of mixtures of Ta_2_O_5_ and MgCO_3_ showed photocatalytic water-decomposition activity without cocatalysts [10]. Due to the inherent coarse-grain structure of MgTa_2_O_6_ powders fabricated by the solid-state reaction, their photocatalytic activity is quite low. With the help of a block polymer P123, a Mg–Ta oxide powder with highly ordered mesopores was previously synthesized [11]. After removal of the P123 template by washing in water, the wormholelike microporous MgTa_2_O_6_ powder, though amorphous, showed enhanced water-decomposition activity when compared to the crystalline MgTa_2_O_6_ prepared by a solid-state reaction. The high activity is ascribed to the thin walls (2.8 nm) separating the mesopores, as the excited electrons and holes only have to travel a short distance to the surface [11]. The use of photocatalysts in the form of thin films avoids the laborious recycling procedure, and this has promoted the development of high-efficiency MgTa_2_O_6_ thin films. One can expect enhanced photocatalytic activity from MgTa_2_O_6_ thin films with well-crystallized nanocrystallites and ordered mesopores, which guarantee the photocatalyst access to the target molecules and also possess a high specific surface area. Unfortunately, because of the high crystallization temperature and low decomposition temperature of most commercially available templates, the fabrication of ordered mesoporous MgTa_2_O_6_ thin films with crystallized walls remains a complex task, and hence detailed photocatalytic-activity studies of such films are scarce.

In particular, suitable well-defined mesoscopic, photocatalytically active metal oxides would be ideal materials for a case study to address the importance of high crystallinity coupled with a well-developed mesostructure. It has been often stated that the performance of mesoporous metal oxides requires both features to be optimized at the same time, but to the best of our knowledge no systematic study has been performed in the field of photocatalysis.

Various oxides with high crystallization temperatures have only recently been synthesized successfully in the form of highly ordered mesoporous thin films, thanks to novel templates such as poly(ethylene-*co*-butylene)-*b*-poly(ethylene oxide), “KLE” (Kraton Liquid -*block*-poly(ethylene oxide)). The KLE template is capable of forming relatively large mesopores and thick walls, and at the same time possesses a high decomposition temperature of up to 400 °C, which facilitates preservation of the ordered mesoporous structure during heating to a high crystallization temperature [12–14]. This work reports the synthesis and photocatalytic characterization of crystallized, mesoporous MgTa_2_O_6_ thin films, obtained through an evaporation-induced self-assembly (EISA) approach, with the help of the KLE template. The photocatalytic activity of the nanoarchitectured MgTa_2_O_6_ thin film was compared both to that achieved by using the BASF Pluronic F127 (*block* copolymer EO_106_–PO_70_–EO_106_), which forms larger mesopores compared to most other commercially available templates, and to a previously reported type of anatase TiO_2_ thin film with ordered mesopores, examples of which are widely used as photocatalysts [15–16]. Recently, a further class of block copolymers with advanced templating properties was introduced, namely poly(isobutylene)-*b*-poly(ethylene oxide) [14,17]. Here, we also used such a polymer, namely “PIB6000”, CH_3_C(CH_3_)_2_(CH_2_C(CH_3_)_2_)_107_CH_2_C(CH_3_)_2_C_6_H_4_O(CH_2_CH_2_O)_100_H, see [17], as an additional structure-directing agent.

As a main motivation, our study addressed the influence of the heat-treatment temperature (i.e., crystallite size) on the photocatalytic activity. Thereby, the influence of the crystallite size and mesostructural organization on the photocatalytic activity was systematically investigated by using dye degradation as a qualitative measure.

## Results and Discussion

### Film characterization

In the following, we focus on mesoporous MgTa_2_O_6_ films templated by the KLE block copolymer. PIB6000 generates films with very similar mesopore structure and crystallinity, with only the average lateral size of the mesopores (ca. 20 nm) being larger than in the case of KLE template films (ca. 15 nm). Since the mesopore structure (spherical mesopores) and also photocatalytical activity of these films are very similar, in the following, mainly KLE-templated films are discussed. Additional results for PIB6000-templated mesoporous films are presented in Supporting Information File 1.

**Figure 1:**
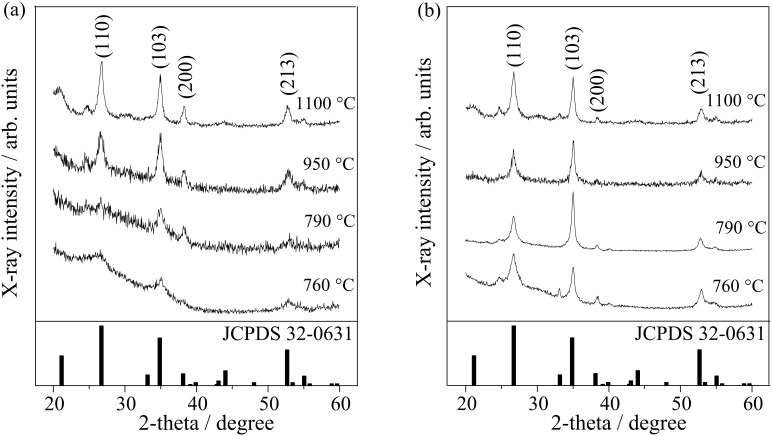
XRD patterns of (a) the KLE-templated mesoporous and (b) the nonporous MgTa_2_O_6_ thin film after thermal treatment at various temperatures.

**Figure 2:**
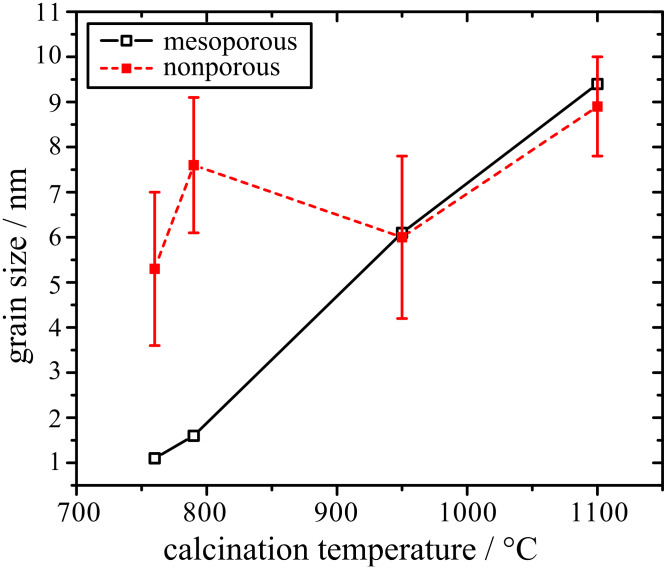
Average grain size and its standard deviation as a measure of anisotropy of MgTa_2_O_6_ in the mesoporous KLE-templated and nonporous films, as a function of the calcination temperature.

Both the mesoporous and the nonporous (nontemplated) films show distinct X-ray diffraction (XRD) reflections corresponding to a tri-rutile structure MgTa_2_O_6_ (JCPDS card 32-0631), only after calcination at temperatures beyond 760 °C, as illustrated in Figure 1. The crystallinity of MgTa_2_O_6_ in the KLE-templated mesoporous film increased with increasing calcination temperature from 760 up to 1100 °C. The average grain size, which was estimated by applying the Rietveld refinement, is shown in Figure 2 as a function of the calcination temperature for both mesoporous and nonporous films. Owing to the lower overall crystallinity of the investigated material and a high background in some of the analyzed XRD patterns, the microstrain part in the size-microstrain procedure within the Rietveld refinement was not refined and was kept to the instrumental values determined by the crystalline standard. The standard was also used for the deconvolution of the instrumental-broadening contribution. Since the initial refinement gave rather high disagreement between the experimental and calculated curves, particularly in the [103] direction, an anisotropic broadening with a larger crystallite size along the [103] direction was assumed. Accordingly, the difference curves were then smooth and all refinements were satisfactory. In Figure 2, vertical error bars represent the distribution interval of crystallite sizes for all reflections, and therefore the error interval is actually a measure of crystal anisotropy. It is obvious that the nonporous crystallites appear with a larger anisotropy (larger grains in [103] direction) compared to their mesoporous counterparts. When the calcination temperature was increased from 760 to 1100 °C the grain size of MgTa_2_O_6_ in the mesoporous film showed a gradual increase from 1.1 to 9.4 nm. When both films were heated to the same temperature of 760 and 790 °C, the crystallinity of MgTa_2_O_6_ in the nonporous film was more pronounced than in the mesoporous one, which can be attributed to the retarding effect of the template and the mesopores on the grain growth.

Grain growth of MgTa_2_O_6_ in the nontemplated film with increasing calcination temperature from 760 up to 1100 °C was not so conspicuous. The grain size only increased from 5.3 to 8.9 nm.

**Figure 3:**
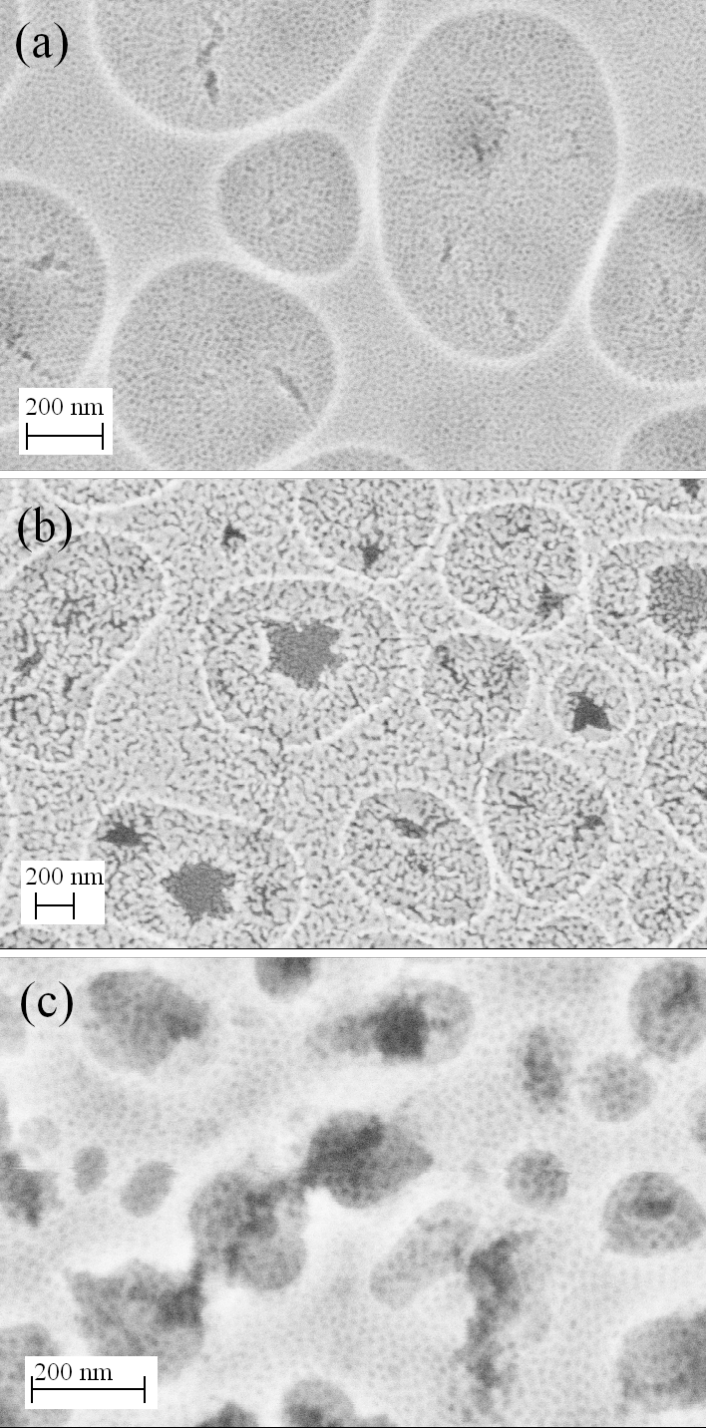
SEM surface morphologies of a mesoporous KLE-templated MgTa_2_O_6_ film after thermal treatment at (a) 760 °C and (b) 1100 °C, and (c) of a mesoporous PIB6000-templated MgTa_2_O_6_ film after thermal treatment at 760 °C.

Figure 3 shows the SEM surface morphology of the MgTa_2_O_6_ thin film subjected to various calcination temperatures. The EISA procedure achieved a crack-free surface with randomly distributed micrometer-sized round concaves, while the film as a whole consisted of highly ordered mesopores. The dual nanoarchitecture remained almost unchanged upon heating to 760 °C, with crystallized MgTa_2_O_6_ appearing within the walls. After being heated to the highest temperature available (1100 °C), conspicuous cracks appeared within the round concaves and the ordered mesopores decayed to a wormlike state. However, the microstructure was still preserved, and most thermally induced cracks were restricted to each concave, that is, they did not coalesce or propagate throughout the film upon heating to 1100 °C. At this temperature, well-crystallized MgTa_2_O_6_ walls were obtained, as seen by XRD.

**Figure 4:**
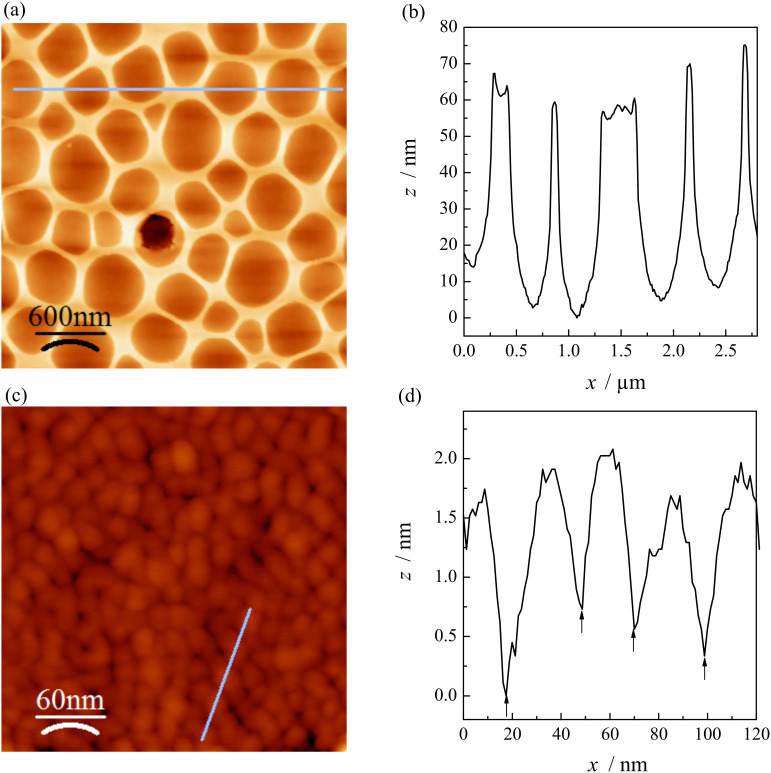
AFM (a, c) morphology of the KLE-templated mesoporous MgTa_2_O_6_ film after calcination at 760 °C. The corresponding profiles along the lines indicated in (a) and (c) are shown in (b) and (d), respectively.

**Figure 5:**
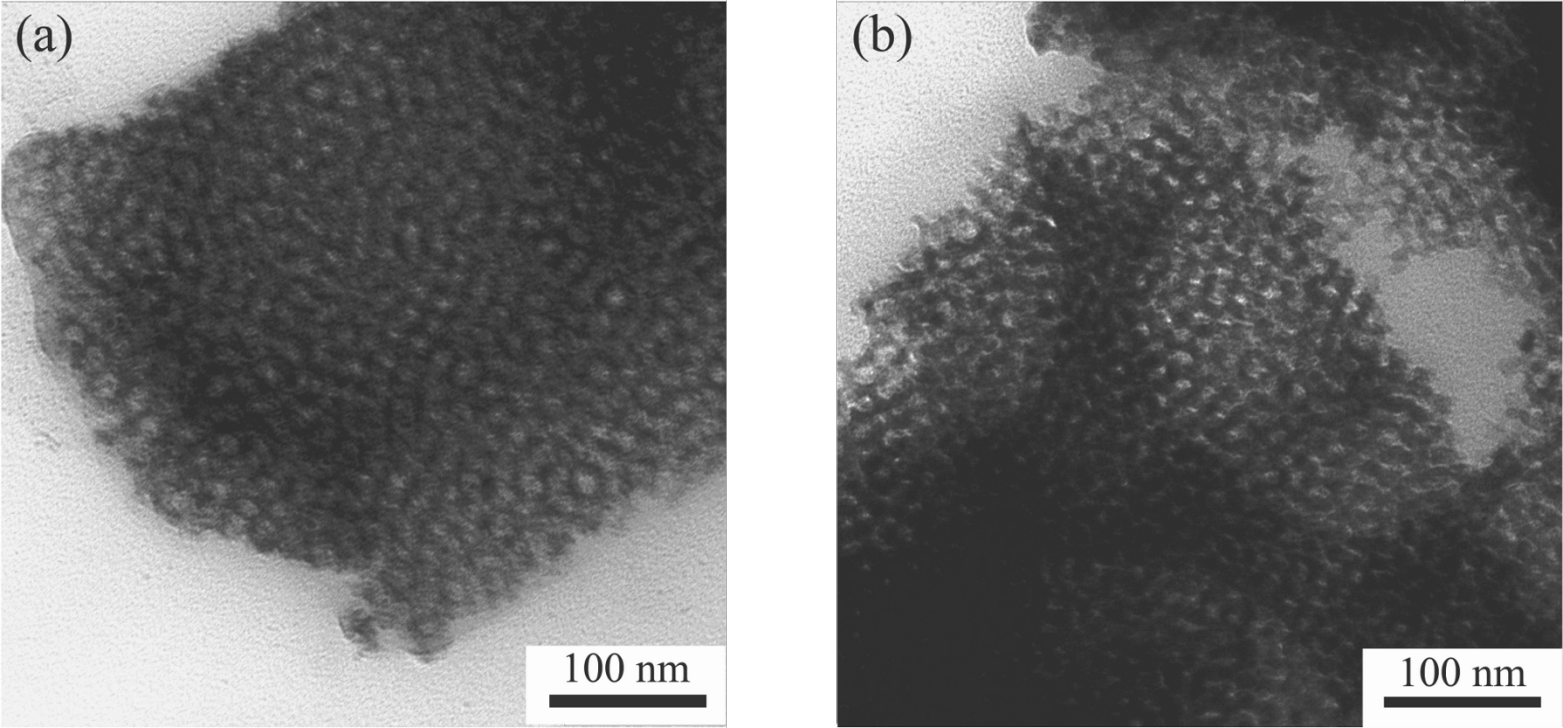
TEM morphology of a fragment scratched off from a mesoporous KLE-templated MgTa_2_O_6_ thin film before (a) and after calcination at 760 °C (b).

Figure 4 illustrates the AFM surface morphology of the MgTa_2_O_6_ film after calcination at 760 °C, which is in agreement with the SEM observation. The corresponding height profile obtained from the AFM image suggests that the depth of the randomly distributed round concaves was ca. 60 nm. The film thickness was estimated to be ca. 200 nm; therefore, the ordered mesoporous MgTa_2_O_6_ film thoroughly covered the Si substrate. The distance between two neighboring mesopores remained constant at ca. 22 nm after calcination at 760 °C. The ordered mesoporous structure was further confirmed by the TEM morphology of flakes scratched off from the MgTa_2_O_6_ film before and after heating at 760 °C, as indicated in Figure 5. From such TEM images a spherical mesopore shape was inferred with an average mesopore diameter of ca. 15 nm. TEM images of PIB6000-templated films are shown in Supporting Information File 1 (Figure S6).

**Figure 6:**
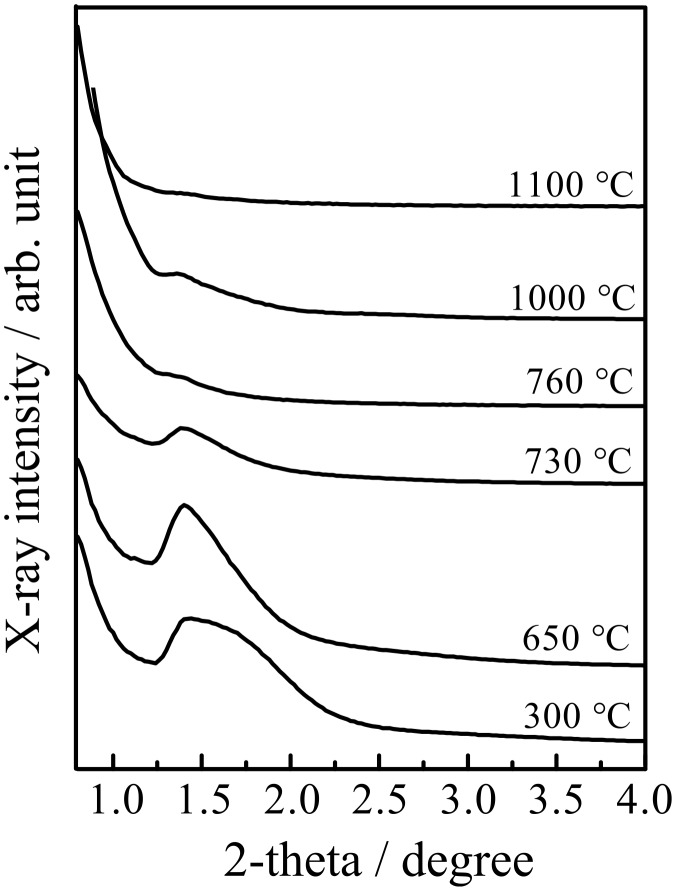
SAXS patterns (measured in symmetric reflection) of the mesoporous KLE-templated MgTa_2_O_6_ thin film after calcination at various temperatures.

Figure 6 shows the SAXS patterns (measured in symmetric reflection with a 1-D detector) of the mesoporous MgTa_2_O_6_ film after calcination at various temperatures. The diffuse SAXS pattern does not allow for an unambiguous determination of the pore morphology. Taking into account previous studies on KLE as a template and the TEM images, the SAXS data can be interpreted in terms of spherical mesopores, although fcc, bcc and hcp structures cannot be distinguished. The scattering maxima are characteristic of ordered mesopores, being assigned as the (110) reflection of a distorted bcc mesoporous structure, and became diffuse as the calcination temperature increased to 760 °C, i.e., the onset of crystallization temperature. However, the SAXS scattering maxima can still be discerned after calcination up to 1000 °C. Therefore, the present ordered mesoporous MgTa_2_O_6_ film possessed a relatively high thermal stability. This property was attributed to the special templating properties of KLE, which forms relatively large micelles during the EISA procedure and hence helps to stabilize the ordered mesopores even after the template has been removed [15–19]. In addition, the randomly distributed round concaves also possibly aid the release of thermal stress, thus helping to maintain the ordered mesopores up to calcination temperatures as high as 1000 °C.

**Figure 7:**
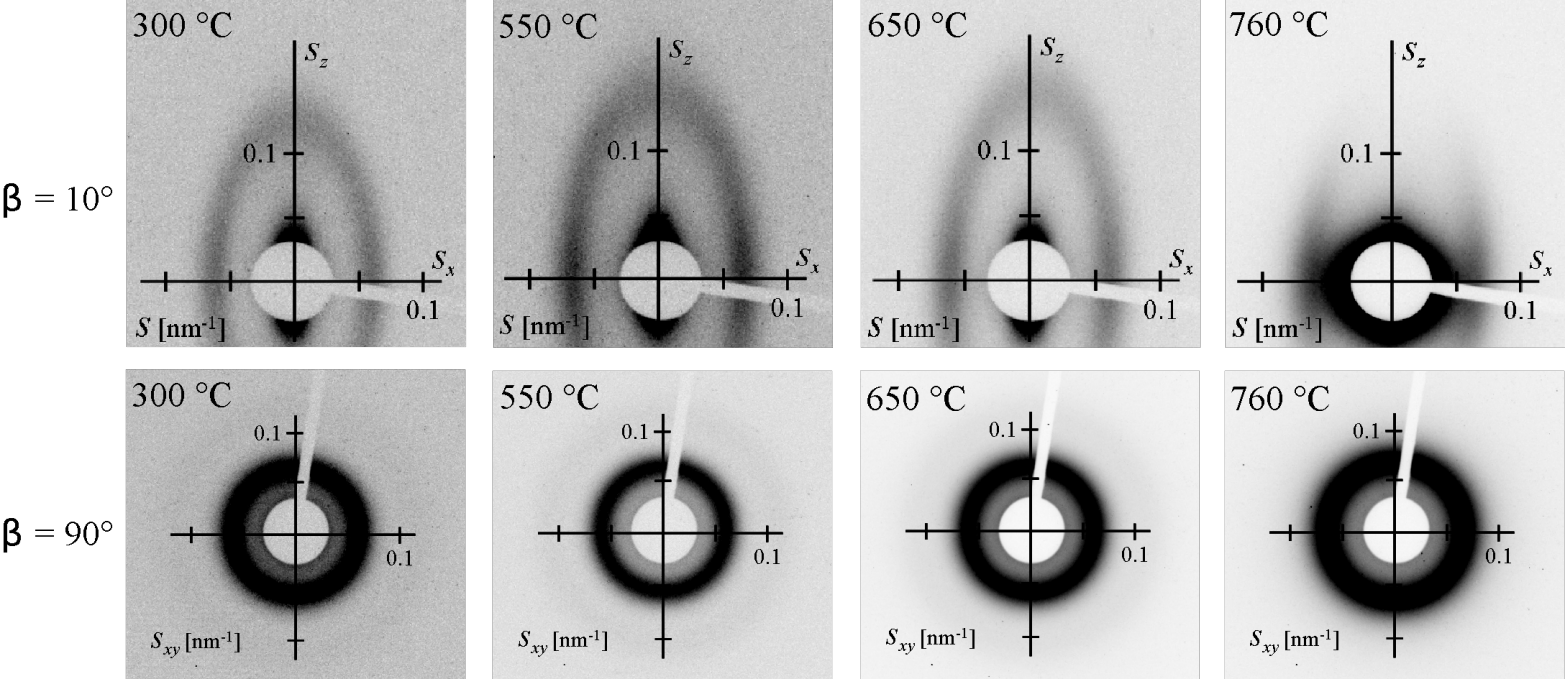
2-D-SAXS patterns of the KLE-templated mesoporous MgTa_2_O_6_ thin film after calcination at various temperatures. The values β = 10° and β = 90° indicate the angles of incidence between the surface of the film and the X-ray beam.

2-D-SAXS patterns of the as-stabilized mesoporous MgTa_2_O_6_ film and of those heated to 550, 650 and 760 °C are shown in Figure 7. Here, the material was synthesized on ultrathin Si wafers (thickness ca. 30 μm), allowing the penetration of the covered wafers by X-rays. The maxima attributable to a bcc mesostructure in [110] orientation relative to the substrate can be discerned in the 2-D patterns measured with an angle of incidence of β = 10°, even after the onset of crystallization [20]. In addition, the 2-D-SAXS patterns became increasingly ellipsoidal, suggesting an anisotropic shrinkage of the mesostructure in the direction normal to the substrate, which has often been observed previously [20]. For the SAXS patterns obtained in transmission geometry (β = 90°) the 1,−1,0 ring, corresponding to a random orientation of mesostructured domains of the bcc mesoporous structure within the plane parallel to the substrate, was observed for all the films. No in-plane contraction in the plane occurred even for a film heated to 760 °C, which is in agreement with the crack-free surface observed by SEM (Figure 3) and AFM (Figure 4). The in-plane *d*-spacing of 22 nm derived from the 2-D-SAXS patterns collected at β = 90° agreed well with the AFM measurement (Figure 4). 2-D-SAXS data of F127- and PIB6000-templated films are shown in Supporting Information File 1 (Figure S5).

The EISA procedure has been widely utilized to synthesize thin films with ordered mesopores, with the help of certain surfactants; however, such round concaves containing ordered mesopores have not been noticed before. As a preliminary result, we ascribe the formation of such a nanoarchitecture to the Mg^2+^ ions in the precursor, which increased the polarity of the ethanol and enhanced the evaporation speed of the solvent. The rapid evaporation produced randomly distributed bubbles, and when these collapsed they left randomly distributed, open, round concaves.

The mesoporosity was additionally investigated by nitrogen physisorption at 77 K (see Supporting Information File 1). Since the amount of material is by far too small for a single film, as an example, four films of PIB6000-templated MgTa_2_O_6_ were deposited on top of each other in order to provide a sufficiently large absolute surface area. These multilayer films were studied by SAXS and SEM to ensure that indeed the multilayer deposition did not disturb the mesostructure. For comparison, multilayers of nontemplated films were studied by nitrogen physisorption.

For such a PIB6000-templated mesoporous multilayer MgTa_2_O_6_ film with four single layers a BET surface area of ca. 260 m^2^ cm^−3^ was observed (after heat treatment at 790 °C), which is typical of mesoporous films with such large mesopores. While the quality of the isotherm is limited, owing to the low amount of material, an average mesopore size of ca. 12 nm can be formally deduced from the BJH approach applied to the adsorption branch. Such a value has to be regarded with care, but nevertheless is consistent with the suggestion of the spherical mesopores being substantially contracted perpendicular to the surface. Thus, nitrogen physisorption provides a well-defined accessible mesopore structure up to high temperatures. A nontemplated film did not exhibit substantial uptake of nitrogen, and no reasonable isotherm was obtained.

### Photocatalytic activity evaluation

**Figure 8:**
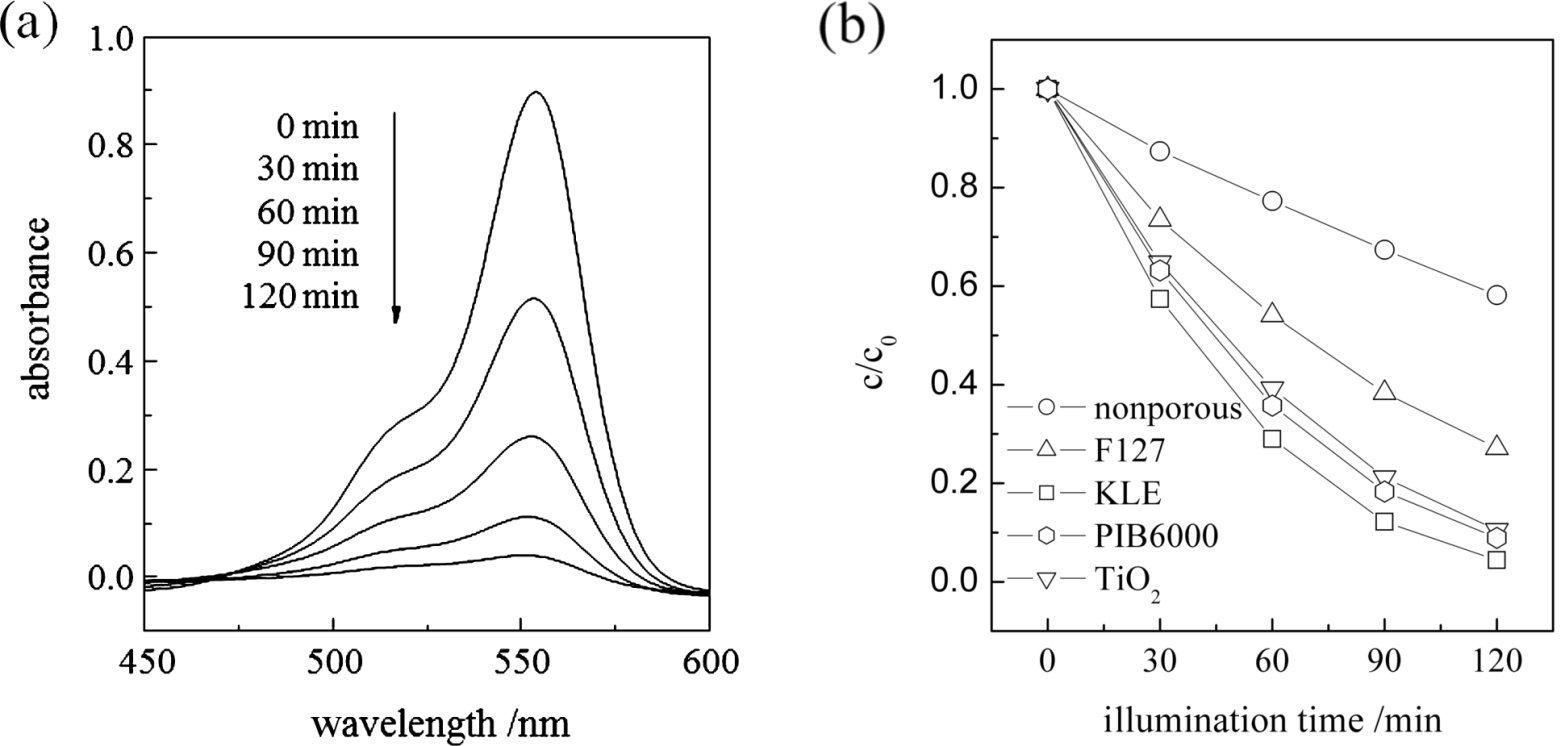
(a) Absorption spectra of RhB after photodegradation for given durations, assisted by the mesoporous KLE-templated MgTa_2_O_6_ thin film after calcination at 790 °C. (b) The RhB concentration as a function of the photodegradation time, assisted by the mesoporous anatase TiO_2_ thin film (“TiO_2_”) after calcination at 550 °C, the KLE-, PIB6000- and F127-templated mesoporous MgTa_2_O_6_ thin film after calcination at 790 °C, as well as the nonporous MgTa_2_O_6_ thin film.

In the absence of the oxide film, the UV irradiation induced no remarkable degradation of rhodamine B (RhB) in water. When the MgTa_2_O_6_ film was exposed to radiation, RhB underwent gradual decomposition depending on the irradiation time. As an example, Figure 8a shows the absorption curve of the aqueous RhB solution as a function of the UV irradiation time in the presence of the KLE-templated MgTa_2_O_6_ film after calcination at 790 °C. The change in RhB concentration as a function of the irradiation time, in the presence of various oxide films, is illustrated in Figure 8b. Here, two oxide films, namely, a mesoporous anatase film and a F127-templated MgTa_2_O_6_ film, were included as references. The fabrication and characterization of the mesoporous anatase film is reported elsewhere [21]. F127-templated MgTa_2_O_6_ films also exhibited an ordered mesoporous structure with concaves, even after calcination at 760 °C; however, the pore-to-pore distance was much smaller and the mesopores were less ordered compared to those templated by KLE. In addition, the round concaves were much smaller and distributed less homogeneously. After calcination, the F127-templated MgTa_2_O_6_ film exhibited XRD patterns corresponding to tri-rutile structure MgTa_2_O_6_ (see Figure S1 for the 2-D-SAXS patterns, Figure S2 for the SEM morphology, and Figure S3 for the XRD pattern in Supporting Information File 1). From Figure 8b it can be seen that, of all the various oxide films, the KLE derived mesoporous MgTa_2_O_6_ film possessed the highest activity in assisting the photodegradation of RhB in water.

Interestingly, PIB6000-templated mesoporous MgTa_2_O_6_ thin film showed a very similar dye-degradation activity as compared with the KLE-templated films.

Photocatalytic degradation of RhB in water roughly follows a pseudo-first-order reaction [22–24],

[1]
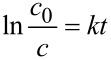


where *c*/*c*_0_ is the normalized RhB concentration, *t* is the illumination time, and *k* is the apparent reaction rate in terms of min^−1^. All of the data demonstrated good linearity for all the curves based on Equation 1 (Figure not shown). The derived reaction rate constant is listed in Table 1. Also included in Table 1 is the so-called turnover frequency (TOF), which defines the ratio of the reaction rate constant to the catalyst content and reflects the intrinsic activity per site of catalysis [25]. The catalyst content in the mesoporous film was calculated assuming a condensed film with a porosity of ca. 30% and a thickness of 200 nm (the densities of MgTa_2_O_6_ [1] and anatase TiO_2_ [24] are 7.2 g cm^−3^ and 3.8 g cm^−3^, respectively), which would overestimate the catalyst content and hence underestimate the TOF value for the mesoporous films. A detailed description of the result in Table 1 will be presented later.

**Table 1:** Reaction rate constant (*k*) and turnover frequency (TOF) of selected photocatalysts.

photocatalyst	*m* (catalyst content) / mmol	*k* / min^−1^	TOF (*k*/*m*) / (mmol·min)^−1^

nonporous MgTa_2_O_6_ film (790 °C)	0.00490	0.00448	0.914
nonporous MgTa_2_O_6_ film (950 °C)	0.00490	0.0175	3.57
KLE-derived MgTa_2_O_6_ film (790 °C)	0.00490	0.0260	5.30
F127-derived MgTa_2_O_6_ film (790 °C)	0.00490	0.0109	2.22
PIB-derived MgTa_2_O_6_ film (790 °C)	0.00490	0.0202	4.12
mesoporous TiO_2_ film (550 °C)	0.0152	0.0203	1.34

**Figure 9:**
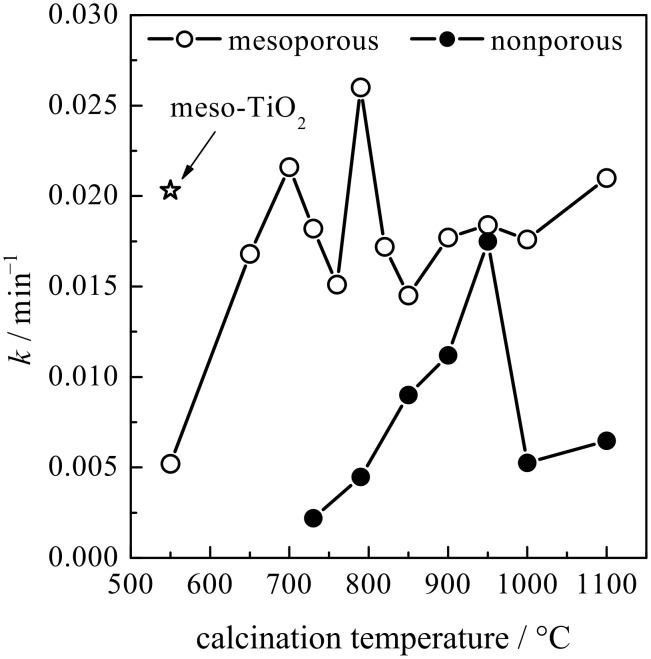
Reaction rate constants of the photodegradation reaction in the presence of the KLE-templated mesoporous and nonporous MgTa_2_O_6_ thin films after thermal treatment at various temperatures. The value for a mesoporous TiO_2_ thin film is represented by a star in the figure for reference.

Figure 9 shows the reaction rate constants of the mesoporous and nonporous MgTa_2_O_6_ films as a function of the calcination temperature. The photocatalytic activity of the nonporous film reached an optimum value of ca. 0.0175 min^−1^ after being heated to 950 °C. However, the evolution in photocatalytic activity for the mesoporous MgTa_2_O_6_ film with increasing calcination temperatures was considerably more complicated. Between 550 °C and 760 °C the photocatalytic activity initially increased to a maximum of 0.0216 min^−1^ at 700 °C, but then decreased as the calcination temperature rose to 760 °C. Interestingly, the photocatalytic activity increased again to a maximum of 0.0260 min^−1^ when the calcination temperature rose from 760 °C to 790 °C. There was a dramatic drop in photocatalytic activity as the temperature increased to 850 °C followed by a gradual increase as the temperature rose to 1100 °C.

Since this peculiar increase and decrease in the activity is one of the most relevant findings of the present study, the measurements were repeated three times. As the results were similar to PIB6000-templated films, Figure 9 shows only one of the films for KLE-templated MgTa_2_O_6_.

Under UV illumination, MgTa_2_O_6_ adsorbs photons with a wavelength shorter than ca. 278 nm and generates electron–hole pairs corresponding to its bandgap of ca. 4.4 eV [10] (see Figure S4, Supporting Information File 1, for the UV–vis diffuse-reflectance spectrum). The photogenerated holes diffuse all the way to the surface where they oxidize the preadsorbed RhB molecules directly, or indirectly by forming hydroxyl radicals, which abstract H atoms from the organics on or near the semiconductor surface [26]. During subsequent calcination, three factors readily affecting the photocatalytic activity changed dramatically in the current investigation, i.e., crystallinity, grain size, and ordered mesopores [27–28]. Defects serve as traps for photogenerated electron–hole pairs [26]; therefore, improved crystallinity prolongs the lifetime of the photogenerated pairs, thus increasing the number of holes that are able to diffuse all the way to the surface to be involved in the photodegradation reaction. Small grains are assumed to facilitate the photocatalytic reaction, as it takes less time for the photogenerated pairs of holes and electrons to migrate all the way to the surface to be involved in the reaction, which thus inhibits the recombination of the pairs [27–28]. Ordered mesopores being accessible from the top favors the diffusion of organic molecules to the oxide surface and at the same time significantly increases the number of catalytically active sites [15]. In a word, improved crystallinity and small grain size of oxides, together with well-ordered mesopores contribute to the photocatalytic activity of semiconductor oxide films with ordered mesoporous structures.

Increasing the calcination temperatures promotes crystallinity, but at the same time causes grain growth and deterioration of the ordered mesoporous structure; consequently, choosing the calcination temperature is always a balancing act. Considering that 760 °C is the onset of crystallization for the present MgTa_2_O_6_ (Figure 1), the fact that the first maximum appeared at 700 °C in the temperature range of 550–760 °C for the mesoporous MgTa_2_O_6_ film can be attributed to the balancing act between the crystallinity and the ordered mesopores. When heated to a temperature tens of degrees below the onset of crystallization, e.g., 700 °C, the decay in the ordered mesopores was not so significant, as can be discerned from the SAXS results shown in Figure 6 and Figure 7. Therefore, the enhanced crystallinity obtained by increasing calcination temperature produced a near linear improvement in the photocatalytic activity at 700 °C. As the calcination temperature approached that of the onset of crystallization, decay in the ordered mesoporous structure became significant due to the further mechanical stress accompanying the amorphous–crystalline phase transformation [29]. There was a decline in photocatalytic activity at the point where the advantages of the increasing crystallinity were outweighed by the disadvantage introduced by the deterioration of the ordered mesopores.

After the onset of crystallization, the negative effect caused by the collapsed mesoporous structure stabilized. In this case, one should expect the gradually increasing crystallinity to produce a gradual increase in photocatalytic activity as calcination temperatures rise. However, the other factor readily affecting the photocatalytic activity of a crystallized oxide, i.e., the grain size, should not be neglected. After the onset of crystallization, the grain size of MgTa_2_O_6_ increased steadily with increasing calcination temperature from 760 °C to 950 °C, as indicated in Figure 2. The negative effect caused by larger grains, in combination with the positive effect originating from the improved crystallinity, resulted in the second maximum of photocatalytic activity at 790 °C in the temperature range of 760–850 °C. The gradual increase in photocatalytic activity of the films after calcination beyond 850 °C can be ascribed to a consistently improved crystallinity and also to the fact that the changes in the grain size and mesopores were not overly significant.

The positive impact of the ordered mesoporous structure on the photocatalytic activity is reinforced by the significantly higher photocatalytic performance of the mesoporous MgTa_2_O_6_ film compared to the nonporous one (Figure 9). In addition, the KLE-templated mesoporous MgTa_2_O_6_ film exhibited higher photocatalytic activity than the F127-templated counterpart at the optimized calcination temperature of 790 °C. In the current investigation, a MgTa_2_O_6_ film with an ordered mesoporous structure was also successfully synthesized by using the commercial F127 template even after calcination at 760 °C; however, the mesopores were much smaller and less ordered. It is not possible to synthesize ordered mesoporous MgTa_2_O_6_ films with crystallized walls by using the other widely used block polymer of P123, because of the even smaller mesopores formed during the EISA procedure [11]. Therefore, the present KLE template contributes greatly to the MgTa_2_O_6_ film with microstructures favoring the photocatalytic property.

Besides the ordered mesoporous structure, the randomly distributed round concave areas with depths of ca. 60 nm might further contribute to the photocatalytic activity. Compared to the generally achieved ordered mesoporous structure with a smooth surface, such a 3-D nanoarchitecture enlarged the surface area of MgTa_2_O_6_ exposed to the RhB solution. Meanwhile, as evidenced from Figure 3, cracks that initiated during the calcination procedure, due either to the thermal mismatch between the MgTa_2_O_6_ film and the substrate or to the mechanical stress originating from the amorphous–crystalline transformation, were effectively confined within the round concaves, which improved the interfacial strength of the thin film.

The present nanoarchitectured mesoporous MgTa_2_O_6_ film possessed an optimized photocatalytic activity significantly higher than that of the ordered mesoporous anatase film. When considering the TOF value, the nanoarchitectured MgTa_2_O_6_ film calcinated at 790 °C possessed a photocatalytic activity ca. four times that of the mesoporous anatase film (Table 1). Although MgTa_2_O_6_ powders have been reported to possess the ability to assist photoinduced water-splitting, their activities are not promising [10–11]. The successful synthesis of the present novel nanoarchitectured MgTa_2_O_6_ film significantly promoted the potential property of MgTa_2_O_6_ as a photocatalyst, which we believe could find practical applications in heterogeneous photocatalysts and solar-induced water splitting.

## Conclusion

Well-crystallized MgTa_2_O_6_ films with ordered mesoporous structures, imposed by randomly but homogeneously distributed round concaves, were synthesized through evaporation-induced self-assembly followed by calcination at temperatures above 760 °C. Such an advanced mesostructure can be achieved by special block copolymers such as KLE or polymers of the poly(isobutylene)-*b*-poly(ethylene oxide) type, which show similar properties in terms of structure and photocatalysis (see Supporting Information File 1). With increasing calcination temperature up to 1100 °C, two maxima in the photocatalytic activity for the MgTa_2_O_6_ film were achieved as a result of the combined effects of increasing crystallinity, increasing grain size, and deterioration of the ordered mesopores. The MgTa_2_O_6_ film with a novel nanoarchitecture possessed an enhanced ability to assist photodegradation of rhodamine B in water under UV irradiation. The optimal photocatalytic activity, when evaluated in terms of turnover frequency, was about four times that of previously reported anatase films with ordered mesopores [15] and even exceeded the photocatalytic performance of such films doped with other elements (see, e.g., [30] and references therein).

In conclusion, our work represents a show-case study regarding the interplay of crystallinity/crystallite size and mesoscopic order. The combination of photocatalytical characterization with comprehensive, temperature-dependent structural analysis proved that mesoporosity itself is not sufficient to endow metal oxides with advanced physicochemical performance.

## Experimental section

### Film fabrication

MgCl_2_ (47 mg; 99.99%, Aldrich) and Ta(OC_2_H_5_)_5_ (500 mg; 99.98%, Aldrich) were dissolved separately in ethanol (2 g) under magnetic stirring. After the addition of concentrated HCl (1.5 g) to the Ta(OC_2_H_5_)_5_ solution, the MgCl_2_ ethanolic solution was added dropwise. Finally, an isotropic solution consisting of the KLE template (100 mg; or 100 mg of the polymer F127 or PIB6000), ethanol (2 g) and tetrahydrofuran (1 g) was added, and the final precursor was stirred for a further 6–10 h before dip-coating. This amount of block copolymers was found to be optimum with respect to the mesostructural organization. The MgTa_2_O_6_ thin films were deposited on Si wafers by dip coating at a controlled relative humidity of 12% with a constant withdrawal speed of 6.5 mm s^−1^, and then dried at 80 °C for 1 h in air and maintained at 300 °C overnight in order to stabilize the mesoporous structure. The subsequent calcination was conducted by heating to the desired temperatures at a rate of 5 K min^−1^. The samples were then removed immediately and allowed to cool down rapidly. The nonporous MgTa_2_O_6_ film was fabricated in a similar procedure except that no template was added in the precursor.

### Film characterization

Scanning electron microscopy (SEM) observations were performed in a LEO440 instrument with an acceleration voltage of 3.0 kV. Atomic force microscopy (AFM) investigations were conducted with a Nanoscope III, Digital Instruments, in tapping mode. Transmission electron microscopy (TEM) images were taken with a Zeiss EM 912Ω instrument at an acceleration voltage of 120 kV. The small-angle X-ray scattering (SAXS) and X-ray diffraction (XRD) measurements were performed in a Bruker D8 diffractometer with an accelerating voltage of 40 kV and a current of 40 mA, with Cu K_α_ radiation. The 2-D-SAXS measurements were carried out by using a Nonius rotating anode setup (Cu K_α_ radiation with λ = 0.154 nm) featuring a three-pinhole collimation system and a MAR CCD area detector, with a sample-to-detector distance of 750 mm. The angle β between the incident beam and the substrate was set to 10° or 90°. Ultrathin Si wafers with a thickness of ca. 30 μm were used for the 2-D-SAXS measurement.

### Photocatalytic activity measurement

Rhodamine B (RhB), a xanthene dye molecule, was used as a probe to evaluate the photocatalytic activity of the thin films [22]. For each test, 33 mL RhB aqueous solution with an initial concentration of 0.01 mmol L^−1^ was illuminated with an 8 W UV lamp (λ_max_ = 254 nm, CAMAG, Germany) in the presence of the thin films with a total exposure surface area of ca. 16 cm^2^. The distance between the lamp and the film was ca. 2 cm. The solution was stirred continuously and exposed to air during each run of the photocatalytic reaction for up to 120 min, interrupted at an interval of 30 min to monitor the change in RhB concentration. The relative concentration *c*/*c*_0_ of RhB was determined by normalizing the absorption of the solution to that of the initial one (*A*/*A*_0_) after a given reaction duration. Monitoring was conducted with a UV–vis spectrophotometer (UVIKON 931, Kontron Instruments, Switzerland) at a fixed wavelength of 554 nm, using a quartz cuvette of 1 cm as the optical path length.

## Supporting Information

File 1 Additional Figures.
