# Supplementary material for: Mesoporous MgTa2O6 thin films with enhanced photocatalytic activity: On the interplay between crystallinity and mesostructure
Source: Beilstein J Nanotechnol. 2012 Feb 13;3:123–33. doi: 10.3762/bjnano.3.13 (PMC3304311; doi:10.3762/bjnano.3.13)
Supplement: File 1 — Additional Figures. [file Beilstein_J_Nanotechnol-03-123-s001.pdf]

Supporting Information

for

Mesoporous $MgTa_2O_6$ thin films with enhanced photocatalytic activity: On the interplay between crystallinity and mesostructure

Jin-Ming Wu[1], Igor Djerdj[2], Till von Graberg,[3] Bernd M. Smarsly*[3]

Address: [1]Department of Materials Science and Engineering, Zhejiang University, Hangzhou 310027, China, [2]Ruđer Bošković Institute, Bijenička 54, 10000 Zagreb, Croatia, and [3]University of Giessen, Institute of Physical Chemistry, Heinrich-Buff-Ring 58, D-35392 Giessen, Germany

Email: Bernd M. Smarsly* - bernd.smarsly@phys.chemie.uni-giessen.de
* Corresponding author

Additional Figures

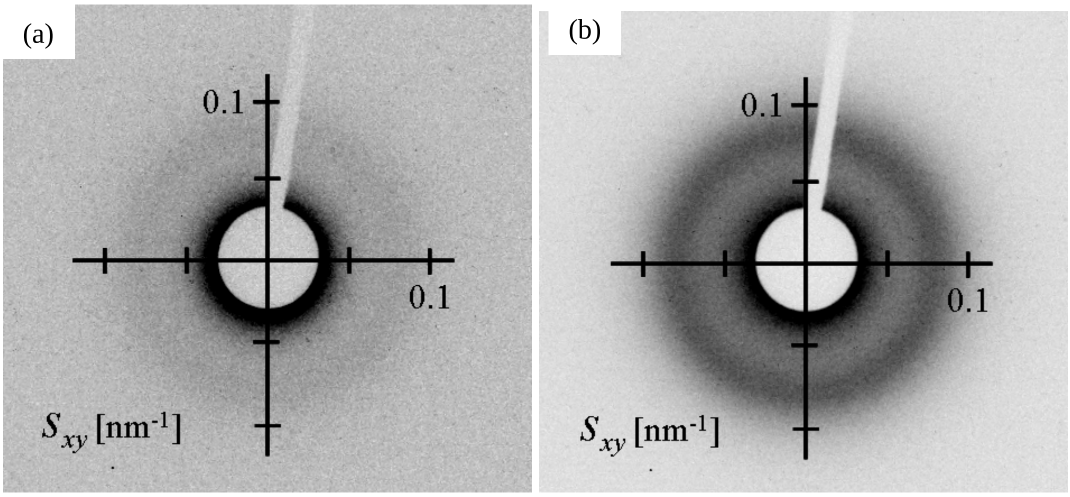

Figure S1: 2D-SAXS patterns of the F127 templated mesoporous $MgTa_2O_6$ thin film before (a) and after thermal treatment at 760 °C (b). β = 90°.

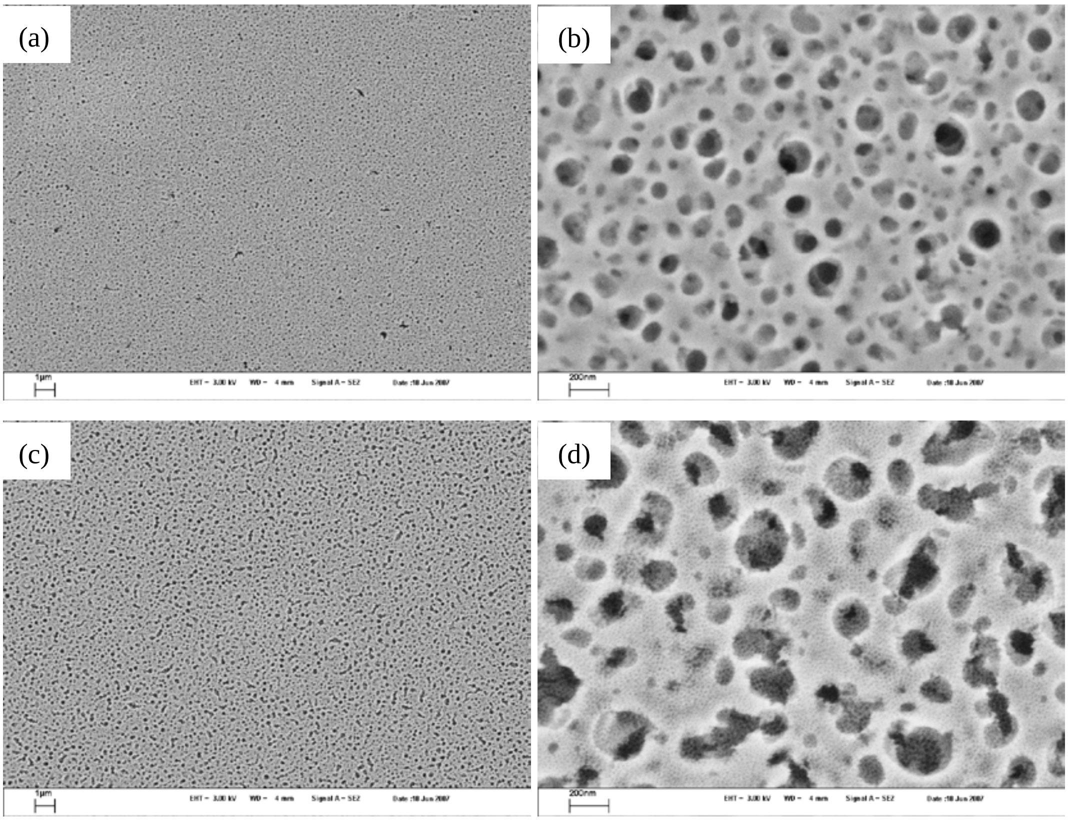

Figure S2: SEM surface morphology of the F127 templated mesoporous $MgTa_2O_6$ thin film before (a, b) and after thermal treatment at 760 °C (c, d).

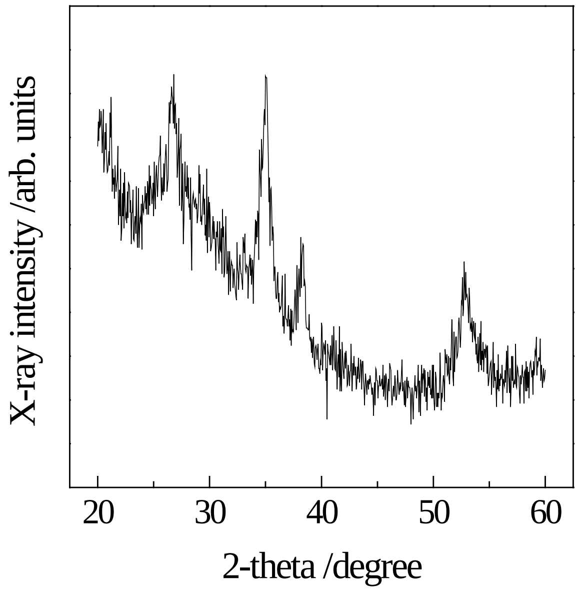

Figure S3: WAXS pattern of the F127 templated mesoporous $MgTa_2O_6$ thin film after calcination at 790 °C.

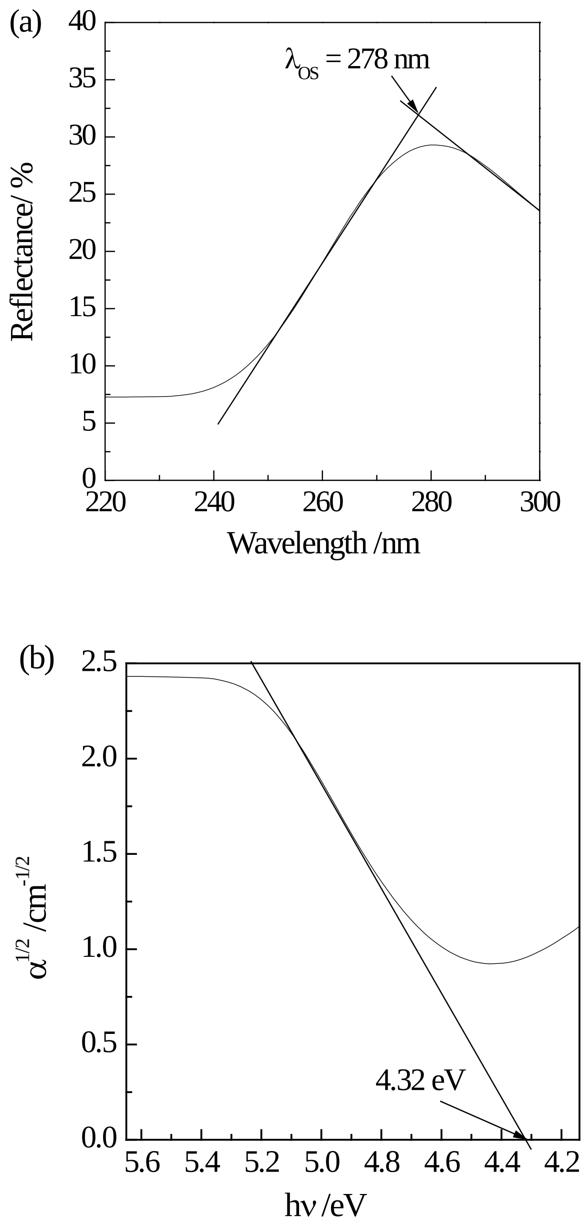

Figure S4: (a) UV–vis diffuse reflectance spectrum of the KLE templated mesoporous $MgTa_2O_6$ thin film after calcination at 790 °C. (b) Replotting of (a) in the $\alpha^{1/2} \sim h\nu$ coordinate to evaluate the corresponding band gap, assuming an indirect transition between bands. The spectrum was collected using a UV–vis near-infrared spectrometer (UV-3150, Shimadzu, Japan).

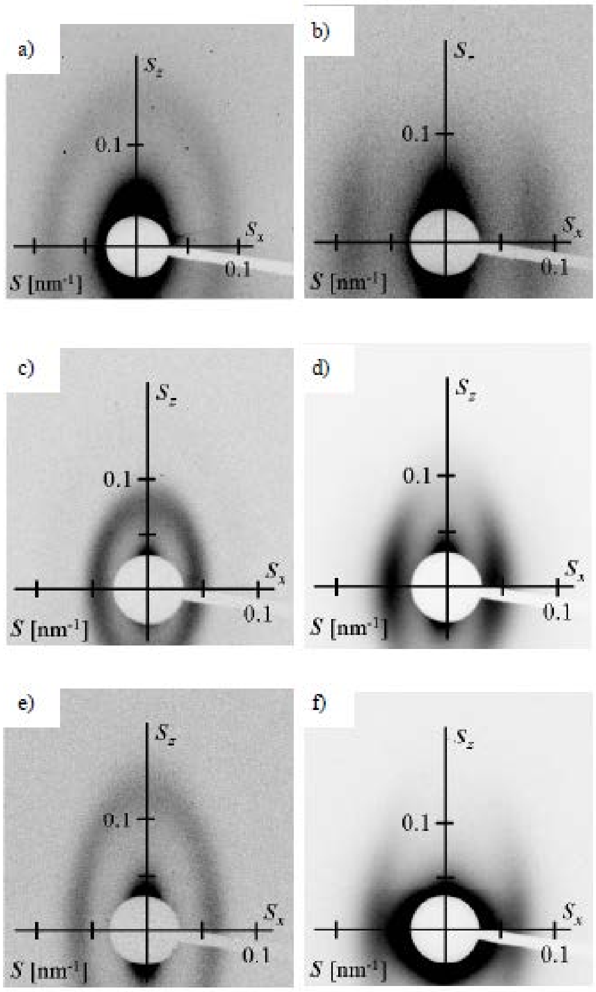

Figure S5: 2-D-SAXS patterns of the mesoporous $MgTa_2O_6$ thin film before and after calcination at 760 °C. Templates: (a, b) F127; (c, d) PIB6000, (e, f) KLE.

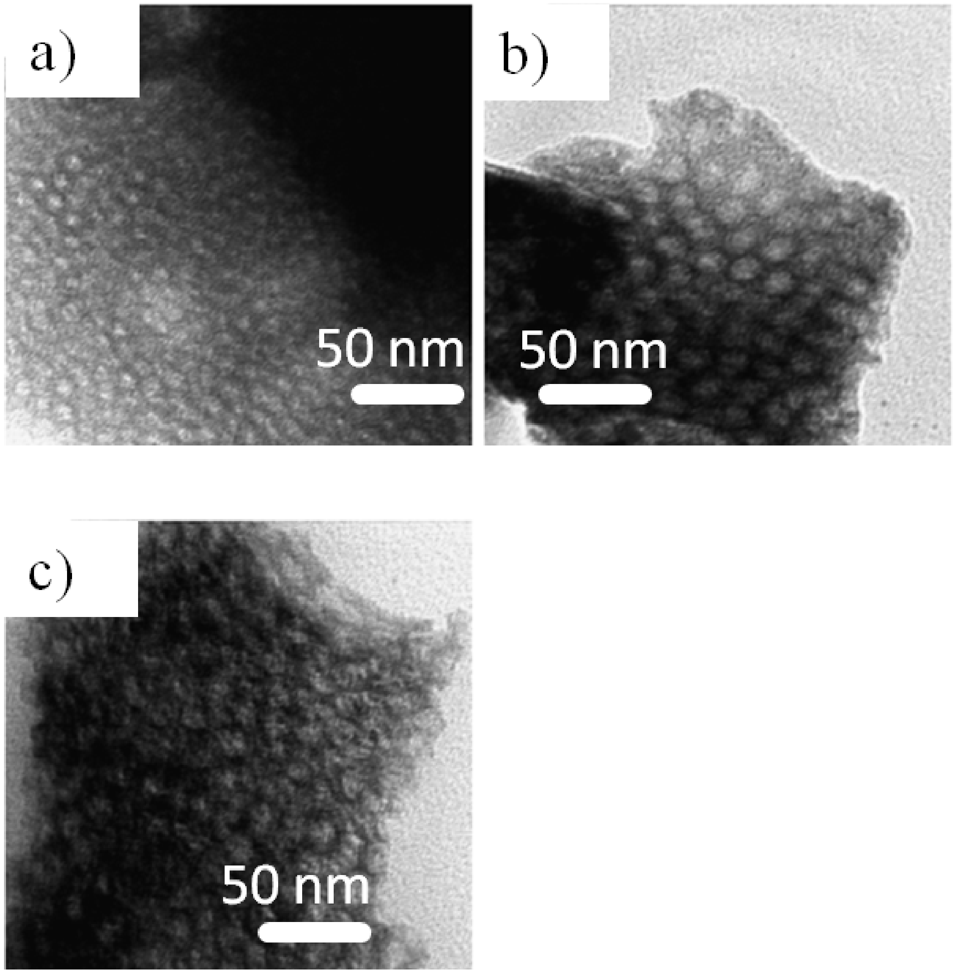

Figure S6: TEM morphology of the mesoporous $MgTa_2O_6$ thin film fabricated using templates of (a) F127, (b) PIB6000 and (c) KLE. All films were treated at 760 °C.

Physisorption analysis of PIB6000 templated mesoporous $MgTa_2O_6$ multilayer films:

Multilayer films for nitrogen physisorption were produced according to the following method. Thin Si-wafers polished on both sides were used for dip-coating at low relative humidity. Afterwards, films were kept at 140 °C for 15 min and then heated to 300 °C within 45 min. After 30 min at 300 °C, the films were removed from the furnace and used for dip-coating again. This procedure was applied until four layers were deposited. Then, the films were heated again to 300 °C and kept there for 12 h. With a temperature ramp of 5 °C min^{-1} the samples were calcinated up to 790 °C to give 550 nm thick films of $MgTa_2O_6$. The total coated area was estimated to be 42.6 cm^2 resulting in a total volume of the films of 2.3 mm^3. The total surface area was then calculated, from N_2-sorption using the BET method, to be 0.603 m^2. This value was divided by the total volume of the films to give a specific surface of 257.4 $m^2\,cm^{-3}$.

Although the shape of the isotherm obtained for $MgTa_2O_6$ does not suit any IUPAC classification, the total surface area derived by the BET method represents a reasonable surface area for mesoporous materials. It should be taken into account that multilayers do not feature the homogeneous and smooth surfaces of single-layered films. The kink in the adsorption branch is due to problems with the equilibration, similarly, the fact that the adsorption and desorption branches do not fall on top of each other points to nonequilibrium effects. Moreover, the precursor solution of this ternary oxide tends toward inhomogeneous evaporation leading to a hindered EISA process. Nevertheless, the shape of the isotherm looks reasonable and speaks for a defined mesoporous material. From SEM imaging of the fourth layer, it can be concluded that a highly mesoporous material is obtained (see Figure S8), indicating that the multilayer film still possesses a well-defined mesostructure. This underlines the value of the specific surface obtained from physisorption.

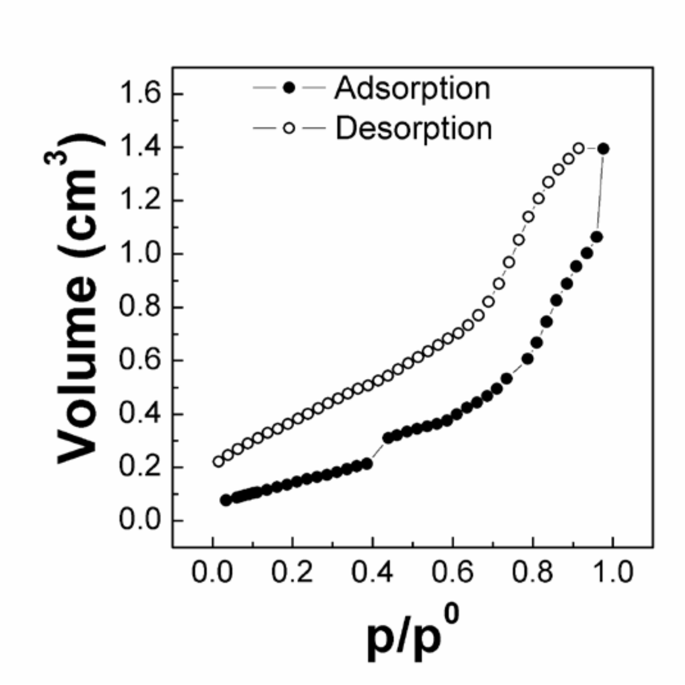

Figure S7: Nitrogen physisorption data for $MgTa_2O_6$ multilayer films.

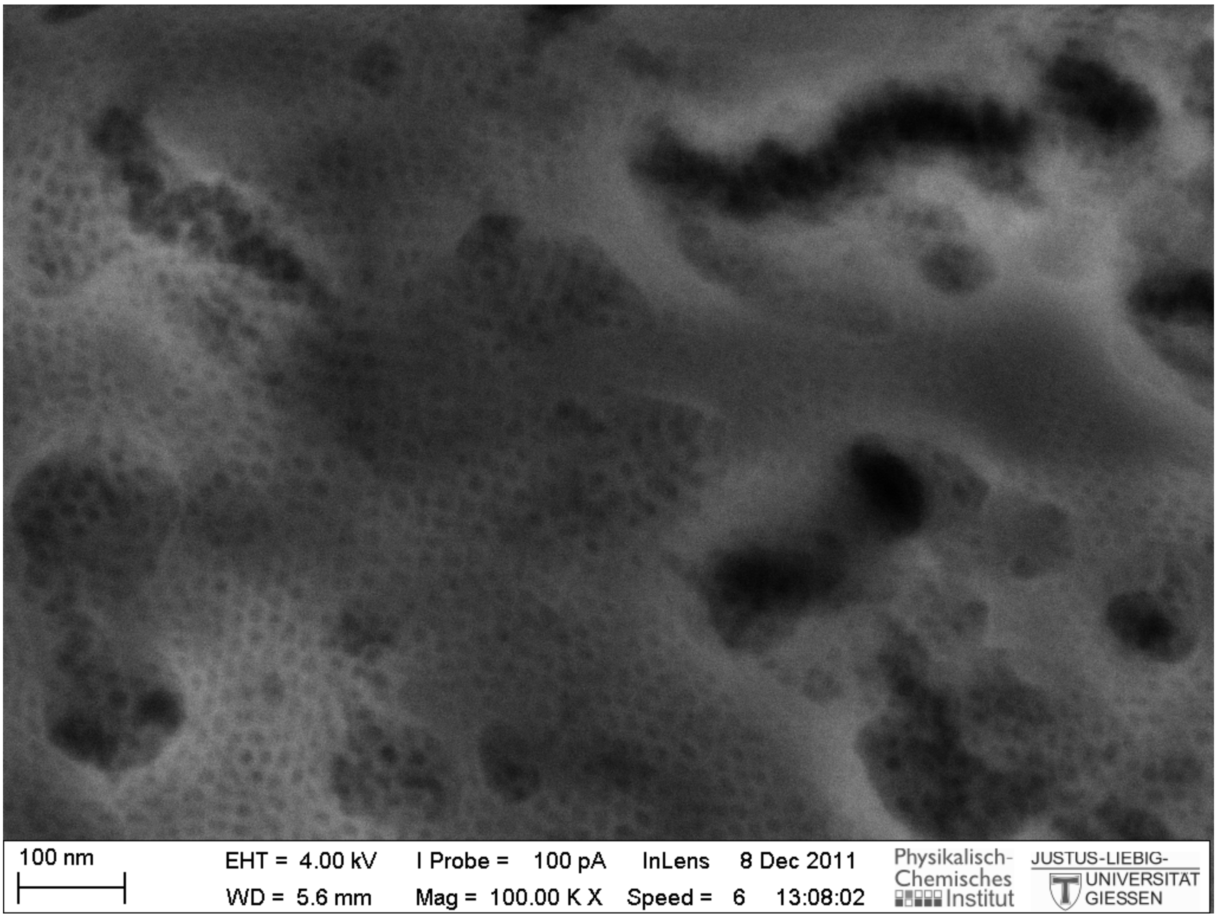

Figure S8: SEM image of the fourth layer of a PIB6000 templated mesoporous multilayer $MgTa_2O_6$ film used for physisorption with nitrogen at 77 K.

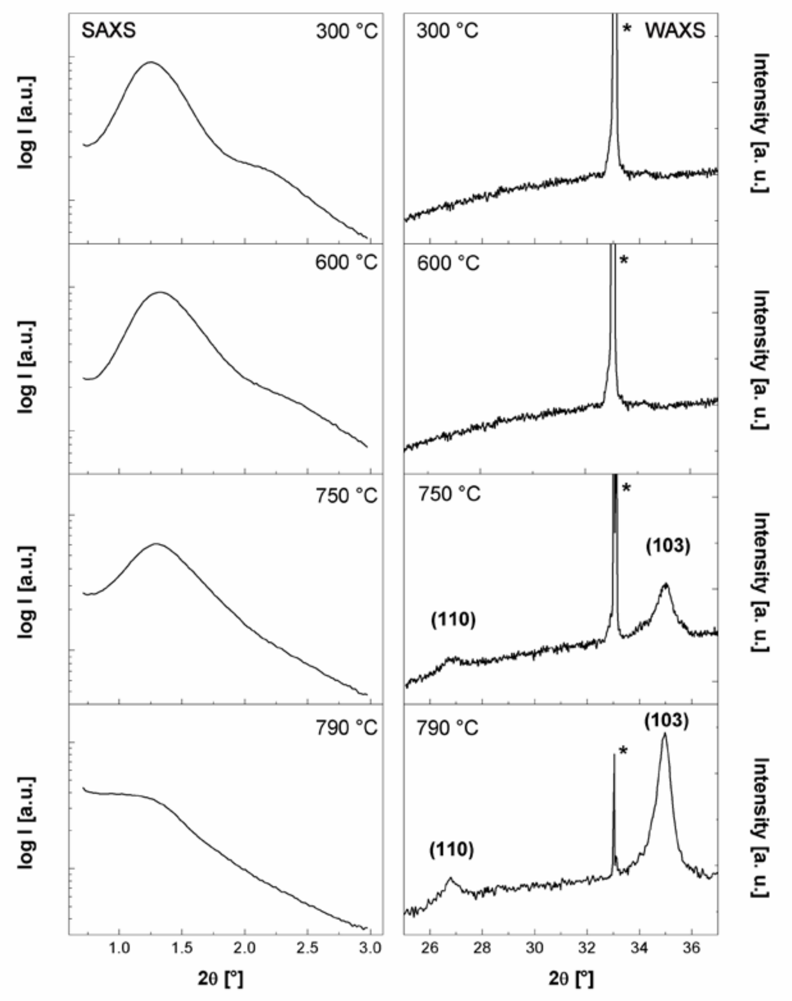

Figure S9: X-ray data of PIB6000 templated multilayer films showing the evolution of the mesostructure by means of SAXS (left) and of the crystallite size by XRD (right), in dependence of the temperature. While between 750 °C and 790 °C the crystallite size increases, the order of the mesostructure starts to collapse.
